# A New Approach Using BMI and FMI as Predictors of Cardio-Vascular Risk Factors among Mexican Young Adults

**DOI:** 10.3390/ejihpe13100146

**Published:** 2023-09-27

**Authors:** Rocío Guadalupe Salinas-Mandujano, Rosalía Reynoso-Camacho, Luis Miguel Salgado, Minerva Ramos-Gomez, Iza F. Pérez-Ramírez, Adriana Aguilar-Galarza, Ulisses Moreno-Celis, Miriam Aracely Anaya-Loyola

**Affiliations:** 1 Department of Food Science, School of Chemistry, Autonomous University of Queretaro, C. U., Cerro de las Campanas, S/N, Queretaro 76010, Mexico; rociosam@hotmail.com (R.G.S.-M.);; 2 Centro de Investigación en Ciencia Aplicada y Tecnología Avanzada, Instituto Politécnico Nacional, Queretaro 76090, Mexico; 3 Department of Studies in Human Nutrition, School of Natural Science, Autonomous University of Queretaro, Av. de las Ciencias, S/N., Juriquilla, Delegación Santa Rosa Jauregui, Queretaro 76230, Mexico

**Keywords:** anthropometric measures, body composition measures, obesity, cardiovascular risk, ROC curve

## Abstract

The study aimed to identify accurate cut-off points for waist circumference (WC), body fat percentage (BF%), body mass index (BMI), fat mass index (FMI), and fat-free mass index (FFMI), and to determine their effective accuracy to predict cardiovascular risk factors (CVRFs) among Mexican young adults. A cross-sectional study was conducted among 1730 Mexican young adults. Adiposity measures and CVRFs were assessed under fasting conditions. The optimal cut-off points were assessed using the receiver operating characteristic curve (ROC). Age-adjusted odds ratios (OR) were used to assess the associations between anthropometric measurements and CVRFs. The cut-off values found, in females and males, respectively, for high WC (≥72.3 and ≥84.9), high BF% (≥30 and ≥22.6), high BMI (≥23.7 and ≥24.4), high FMI (≥7.1 and ≥5.5), and low FFMI (≤16 and ≤18.9) differ from those set by current guidelines. High BMI in women, and high FMI in men, assessed by the 50th percentile, had the best discriminatory power in detecting CVRFs, especially high triglycerides (OR: 3.07, CI: 2.21–4.27 and OR: 3.05, CI: 2.28–4.08, respectively). Therefore, these results suggest that BMI and FMI measures should be used to improve the screening of CVRFs in Mexican young adults.

## 1. Introduction

Obesity is a chronic disease that is increasing in prevalence and is now considered to be a global epidemic [1]. There is a vast amount of data supporting an increased risk of cardiovascular disease (CVD) morbidity and mortality associated with excessive body weight [2]. Although a positive relationship has been established between chronic diseases and body mass index (BMI) [3], some limitations remain to be considered, since BMI lacks discernment between fat and muscle mass [4]. Hence, other measures of adiposity, like waist circumference (WC) and body fat percentage (BF%) [5], as well as body composition measurements, like fat mass index (FMI) and fat-free mass index (FFMI) [6], have been used in clinical diagnosis due to their association with cardiovascular risk factors (CVRFs). Nonetheless, there is still controversy about which anthropometric measures most accurately predict CVRFs since the association between adiposity and cardiovascular health might be influenced by ethnic, sex, and age variations [7].

Additionally, studies on different ethnicities and age groups have shown different cut-off values of anthropometric measures in predicting CVRFs [8,9,10,11,12,13,14,15,16,17]. These findings have suggested that the proposed cut-off values of international organizations to define obesity may be not appropriate for all ethnic and age groups. According to Macias et al. [18], the international BF%, BMI, and WC cut-off points had very low specificity for identifying obesity in Mexican adults (median = 41 years old), which results in a very low accurate identification of CVRFs. Therefore, in agreement with the World Health Organization (WHO), the predictive power of anthropometric measures and their appropriate cut-off points should be established for different populations [19].

To the best of our knowledge, there is no information about accurate cut-off points for adiposity estimation, nor about the analysis of adiposity measurements as predictors of CVRFs in Mexican young adults. This is the first study systematically reporting the abilities of different cut-off points of anthropometric and body composition measures in predicting CVRFs among Mexican young adults.

The aim of our study was to identify accurate cut-off points for WC, BF%, BMI, FMI, and FFMI, as well as to determine their effective accuracy in predicting CVRFs in Mexican young adults. The hypothesis tested was that body composition measures, such as BF%, FMI, and FFMI, may be better associated with CVRFs compared to BMI among Mexican young adults. To test this hypothesis, anthropometric and body composition measures were determined, and multiple statistical analyses were employed regarding the association of these measures with CVRFs, which would help to implement preventive measures against CVRFs.

## 2. Materials and Methods

### 2.1. Study Population

This is a cross-sectional study involving students from the Autonomous University of Queretaro (UAQ, Queretaro-Mexico), who were participating in the University Health promotion program SU-SALUD-UAQ between August 2014 and February 2016. Subjects (n = 1856) were recruited by a non-probabilistic sample, including all the freshmen attending the Autonomous University of Queretaro, who voluntarily agreed to participate in the study. All participants provided written informed consent, in accordance with the Declaration of Helsinki, before the beginning of research procedures. The study was approved by the Ethics Committee of the School of Natural Sciences of the Autonomous University of Queretaro (23FCN, 2014).

Inclusion criteria were being students in the first year of school and being between 18 and 25 years old. We excluded 126 subjects with clinical evidence of infectious disease, pregnancy or lactation, use of prosthesis, and lack of any body segment or biochemical measurements. The final sample consisted of 1730 Mexican young adults (848 women and 882 men), assigned following an external examination of body characteristics.

### 2.2. Anthropometric and Body Composition Measurements, and Blood Pressure

Students were invited in fasting conditions to the Nutrition Clinic to collect their anthropometric measurements including weight, height, BF%, and WC. A well-trained anthropometrist performed all the measures. Body weight and BF% were measured simultaneously using a digital scale Body Composition Analyzed X-Scan plus II (Jawon Medical Mod 514 Co., Ltd., Seoul, Republic of Korea). Height was measured using a mechanic stadiometer (SECA Mod 216, Hamburg, Germany). WC was measured midway between the lowest rib and the iliac crest using flexible fiberglass measuring bands (SECA Mod 200, Hamburg, Germany). BMI was calculated as the body weight divided by squared height (kg/m^2^). Fat-free mass (FFM) and fat mass (FM) indexes were estimated according to the following mathematical expressions: FMI = fat mass/height^2^ (kg/m^2^) and FFMI = fat-free mass/height^2^ (kg/m^2^) [20]. Systolic and diastolic blood pressures were obtained from the right arm in a sitting position using a mercurial sphygmomanometer (Medimetrics Mod 5881) and were recorded as the mean of three measurements.

### 2.3. Biochemical Measurements

Fasting blood samples were collected by venipuncture from the arms of all students. After blood was drawn, the samples were centrifuged separately and immediately frozen at −80 °C for further analysis. Triglycerides (TG), total cholesterol (TC), high-density lipoprotein cholesterol (HDL-c), low-density lipoprotein cholesterol (LDL-c), and glucose (GLC) were measured using enzymatic-colorimetric kits (Spinreact, Girona, Cataluña, Spain) in a Mindray BS120 biochemical automatized analyzer (Shenzhen, China).

### 2.4. Definition of Cardiovascular Risk Factors

Reference values were defined according to the guidelines of ATP III (2002) [21], as follows: high blood pressure (BP) (≥130/85 mmHg), high GLC (≥100 mg/dL), high TG (≥150 mg/dL), low HDL-c (≤50 mg/dL in women and ≤40 mg/dL in men), high TC (≥200 mg/dL), and high LDL-c (≥130 mg/dL).

### 2.5. Statistics Analysis

Statistical analysis was performed using JMP 10 (North Caroline, USA). Continuous variables are presented as mean ± standard error (SE) and categorical variables as absolute and relative frequencies. All analysis was performed separately for men and women. The Kolmogorov–Smirnov test was used to identify data normality. The continuous variables and categorical variables were compared using the Mann–Whitney U test and the Chi-square (χ^2^) test, respectively, to examine the effects of gender. Statistical significance was set at two-sided *p* < 0.05. Receiver operating characteristic (ROC) curves were developed and the area under the curve (AUC) calculated to obtain the optimum cut-off points to predict CVRFs. The optimal cut-off value was determined by using the point with the highest Youden index (maximum sensitivity + specificity − 1) [22]. Multiple logistic regression analyses were performed to estimate odds ratios (ORs) with 95% confidence intervals (95%CI) of anthropometric and body composition measures as the independent variables for the presence of CVRFs as the dependent variable. Three models were fitted for each measurement by three cut-off points: model 1 in agreement with the cut-off points from current guidelines for WC (≥80 cm in women and ≥94 cm in men), BF% (≥35% in women and ≥25% in men), BMI (≥25 kg/m^2^ in both gender), FMI (≥8.2 kg/m^2^ in women and ≥5.2 kg/m^2^ in men), and FFMI (≤15 kg/m^2^ in women and ≤17 kg/m^2^ in men) [23,24,25]; model 2 consistent with the 50th percentile cut-off points; and model 3 according to the average of optimal cut-off points determined by the Youden index from ROC curves. All models were adjusted by age. Finally, Spearman’s correlation coefficient was used to confirm the statistical relation between anthropometric and body composition measures and CVRFs.

## 3. Results

### 3.1. Characteristics of Study Participants

All mean values for CVRFs were found in the normal range according to the ATP-III guidelines. BMI, FFMI, WC, SBP, DBP, GLC, and TG were statistically significantly higher in men (*p* < 0.0001), and BF%, FMI, and HDL-c were significantly higher in women (*p* < 0.0001). No significant differences were found in age (*p* = 0.680), LDL-c (*p* = 0.399), and TC between both sexes (*p* = 0.387) (Table 1).

### 3.2. Optimal Cut-Off Points of Anthropometric and Body Composition Measures by ROC Curve Analysis

Table 2 shows the optimal cut-off values of anthropometric and body composition variables for predicting the presence of CVRFs in women and men separately. Values for WC were from 63.4 to 86.6 in women and from 81.9 to 91.9 cm in men. For BF%, values ranged from 26.6 to 33.7% in women and from 20.9 to 26.1 in men. For BMI, values were from 21.1 to 27.3 kg/m^2^ in women and from 23.3 to 25.5 kg/m^2^ in men. Regarding FMI and FFMI, values ranged from 6.4 to 8.1 kg/m^2^ and from 15.5 to 16.8 kg/m^2^, respectively, in women, and from 5.0 to 6.6 kg/m^2^ and from 18.6 to 19.1 kg/m^2^, respectively, in men. As expected, when the cut-off values were compared between genders, it was observed that the optimal WC, BMI, and FFMI cut-off values were higher in men while the optimal BF% and FMI cut-off values were higher in women (Table 2). The lower cut-off values in women were observed when they predicted the presence of high TC, high LDL-c, and high GLC, while in men they predicted the presence of high BP and low HDL-c.

### 3.3. Association of Anthropometric and Body Composition Measures and Cardiovascular Risk Factors Using ROC Curve Analyses

The AUC measurements for each anthropometric and body composition measure predicting the presence of CVRFs are also presented in Table 2. Generally, all anthropometric and body composition measures showed larger AUC in men than in women. Both BMI and FMI showed the highest AUC (0.67; 95% CI: 0.61–0.73) when detecting the presence of high TC in men, whereas BMI presented the highest AUC (0.65; 95% CI: 0.61–0.69) when detecting the presence of high TG in women, although differences were small with overlapping 95% CI.

### 3.4. Prevalence of Anthropometric and Body Composition Measures, and Cardiovascular Risk Factors

**Figure 1:**
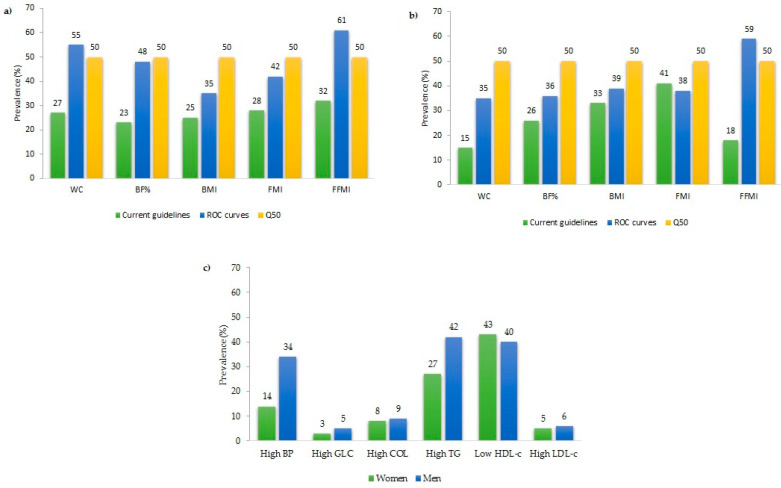
Prevalence of anthropometric and body composition measures according to the cut-off points given by current guidelines, and those obtained from the 50th percentile and averages of ROC curves in women (**a**) and men (**b**), and prevalence of cardiovascular risk factor (**c**). WC, waist circumference; BF%, percentage of body fat; BMI, body mass index; FMI, fat mass index; FFMI, fat-free mass index; BP: blood pressure; GLC, glucose; HDL-c, high-density lipoprotein cholesterol; LDL-c, low-density lipoprotein cholesterol; TG, triglycerides; TC, total cholesterol.

According to cut-off points from current guidelines, women showed a significantly higher prevalence of high WC (27% vs. 15%) and low FFMI (32% vs. 18%), and significantly lower prevalence of BMI (25% vs. 41%) and high FMI (28% vs. 41%) than men, whereas the prevalence of high BF% did not significantly differ between genders (23% in women vs. 26% in men). Nevertheless, when diagnoses were established using the average of optimal cut-off points, determined by the Youden index from ROC curves, women only had a significantly higher prevalence of high WC (55% vs. 35%) and high BF% (48% vs. 36%) than men, while the prevalence of high BMI (35% vs. 39%), high FMI (42% vs. 38%), and low FFMI (61% vs. 59%) were similar in both sexes. Otherwise, it was noticed that the prevalence, established using the 50th percentile, of all anthropometric and body composition variables with the exception of high FMI in men, was higher than that obtained when cut-off points previously cited were used (Figure 1a,b). Furthermore, significant differences were observed in the prevalence of high BP, high GLC, and high TG between men and women. Men presented a higher prevalence of high BP (34% vs. 14%), high GLC (5% vs. 3%), and high TG (42% vs. 27%) compared to women. On the other hand, no significant differences were observed for the prevalence of high TC (9% vs. 8%), low HDL-c (40% vs. 43%), and high LDL-c (6% vs. 5%) between men and women, respectively (Figure 1c).

### 3.5. Odds Ratios of Cardiovascular Risk Factors by Anthropometric and Body Composition Measures

Table 3 and Table 4 summarize the age-adjusted ORs and 95%CI for CVRFs according to the diagnosis of anthropometric and body composition measurements in women and men, respectively. In the first model, the OR was estimated in agreement with the cut-off points for the anthropometric and body composition measures from current guidelines, whereas in the second model it was estimated in agreement with the 50th percentile cut-off points, and in the third model it was estimated according to the average of optimal cut-off points determined by the Youden index from ROC curves.

In all models, women with high BF%, high BMI, high FMI, and high WC had significantly high odds of high TG and low HDL-c. The higher odds of high TG were observed in women with high BMI across the three models (OR: 3.27; 95% CI: 2.29–4.66, OR: 2.87; 95% CI: 2.06–3.99, and OR: 3.07; 95% CI: 2.21–4.27, respectively). However, the odds of low HDL-c were not considered significantly different from each other due to the overlapping 95% CI. In contrast, low odds of high TG and low HDL-c were significantly associated with low FFMI across the three models (OR: 0.45; 95% CI: 0.31–0.66, OR: 0.44; 95% CI: 0.32–0.61, and OR: 0.49; 95% CI: 0.32–0.62, respectively, and OR: 0.45; 95% CI: 0.33–0.62, OR: 0.54; 95% CI: 0.41–0.71, and OR: 0.46; 95% CI: 0.34–0.61, respectively).

Regarding the odds of high BP, it was significantly increased in women with high BF%, FMI, and BMI in the three models, and because of overlapping 95% CI, the odds were not markedly different among the anthropometric and body composition measures.

Moreover, a significant increase in odds of high LDL-c was only observed in model 2 among women with high FMI (OR: 2.25; 95% CI: 1.12–4.53) and high BF% (OR: 2.01; 95% CI: 1.01–3.98). Additionally, high GLC, high TC, and high LDL-c were not related to anthropometric and body composition measures.

Similarly to women, men with high BF%, high BMI, high FMI, and high WC had significantly high odds of high TG, low HDL-c, and high BP, but also with high TC in the three models. The highest odds of high TG were observed in men with high WC (OR: 3.69; 95% CI: 2.44–5.57) in model 1, and in those with high FMI in models 2 and 3 (OR: 2.96; 95% CI: 2.23–3.95, and OR: 3.05; 95% CI: 2.28–4.08, respectively). Furthermore, men with high BF% had the highest odds for low HDL-c (OR: 2.51; 95% CI: 1.84–3.43) in model 1, and those with high FMI in models 2 and 3 (OR:1.95; 95% CI: 1.47–2.57, and OR: 2.17; 95% CI: 1.63–2.88, respectively). In addition, the highest odds of high BP were observed in men with high WC (OR: 3.30; 95% CI: 2.20–4.96) in model 1, and in those with high BMI in models 2 and 3 (OR: 2.72; 95% CI: 2.02–3.37, and OR: 2.64; 95% CI: 1.95–3.55, respectively). Similarly, men with high WC had the highest odds for high TC (OR: 3.24; 95% CI: 1.94–5.41) in model 1, and those with high BMI in models 2 and 3 (OR: 2.52; 95% CI: 1.53–4.16, and OR: 2.94; 95% CI: 1.83–4.73, respectively).

Unlike women, among men high GLC was significantly associated with high BF%, high BMI, and high FMI in the three models as well as low LDL-c with high BMI and high FMI in the three models, with high BF% in models 2 and 3, and with high WC only in model 3. Regarding high GLC, the highest odds were observed in men with high FMI (OR: 2.87; 95% CI: 1.56–5.29) in model 1, with high BMI (OR: 2.19; 95% CI: 1.18–4.08) in model 2, and with high BF% (OR: 2.70; 95% CI: 1.49–4.88) in model 3. On the other hand, the highest odds of high LDL-c were observed in men with high BMI in models 1 and 3 (OR: 2.61; 95% CI: 1.46–4.6,8 and OR: 2.39; 95% CI: 1.33–4.31, respectively), whereas in model 2 the odds were not significantly different among anthropometric and body composition measures due to overlapping 95% CI. Additionally, men with low FFMI showed significantly less risk of having high TG and high BP across the three models, and high GLC, high TC, low HDL-c, and high LDL-c in models 2 and 3. The lowest risk was shown in model 3 for high TG (OR: 0.48; 95% CI: 0.36–0.63), high BP (OR: 0.37; 95% CI: 0.27–0.49), and high TC (OR:0.43; 95% CI: 0.27–0.68), and in model 2 for high LDL-c (OR: 0.47; 95% CI: 0.25–0.87).

### 3.6. Correlations between Anthropometric and Body Composition Measures and Cardiovascular Risk Factors

**Figure 2:**
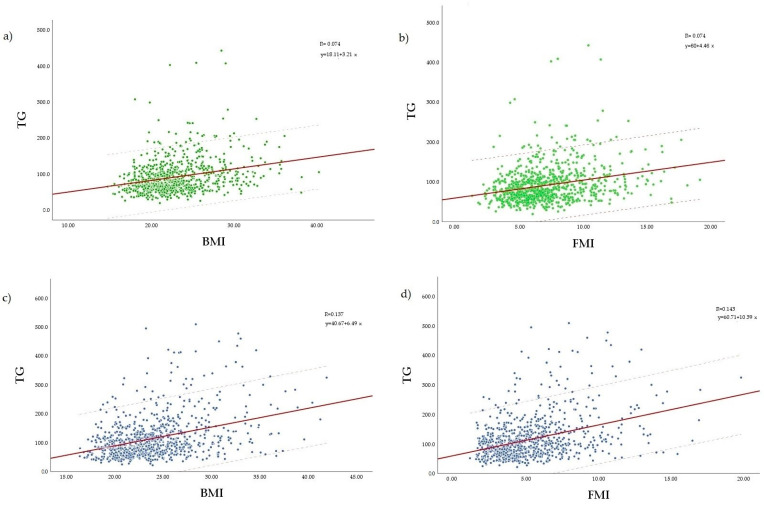
Scatter plot of Spearman’s correlation of body mass index with serum triglycerides in women (**a**) and men (**c**), and fat mass index with serum triglycerides in women (**b**) and men (**d**). BMI, body mass index; FMI, fat mass index; TG, triglycerides.

Spearman’s correlation coefficients were applied to confirm the statistical relation between BMI and FMI, and TG. BMI was positively and significantly correlated with TG in both women (r = 0.286, *p* < 0.0001) and men (r = 0.323, *p* < 0.0001) (Figure 2a,c). FMI also showed a positive and significant correlation with TG in both sexes (r = 0.293, *p* < 0.0001 in women; r = 0.332, *p* < 0.0001 in men) (Figure 2b,d).

### 3.7. Sensitivity and Specificity of Anthropometric and Body Composition Measurements in the Prediction of Cardiovascular Risk Factors

To identify the more accurate cut-off points, used across the three models, of anthropometric and body composition measures for the risk of CVRFs, their sensitivity and specificity were compared. In women (Table 5), the cut-off points proposed in the present study for high BF%, BMI, FMI, and WC had higher sensitivity and specificity than those cited by current guidelines. The highest sensitivity and specificity were shown in the cut-off point obtained from the average of ROC curves for high WC (70% and 54%, respectively, for high TG), followed by those calculated from the 50th percentile for high FMI (68% for high LDL and 51% high TC, respectively), BMI (68% for high TG and 50% for high LDL-c, respectively), and high BF% (65% for high LDL-c and 49% in high GLC, respectively). Only the cut-off point given by current guidelines for low FFMI showed higher sensitivity and specificity than those proposed in this study (79% for high TG and 67% for high LDL-c, respectively). Adversely, in comparison with those proposed in this study, the cut-off points cited by current guidelines had the lowest sensitivities and specificities for high WC (22% for high TC and 21% for high TG, respectively), high BF% (19% for high TC and 17% for low HDL-c, respectively), high BMI (20% for high TC and 19% for low HDL-c, respectively) and high FMI (22% for high TC and 21% for low HDL-c, respectively). Only the cut-off point for low FFMI proposed in the present study from the average of ROC curves, showed lower sensitivity and specificity (34% for high TC and 30% for low HDL-c) than those cited by current guidelines.

Similarly, the cut-off points for high WC, BF%, BMI, and FMI proposed in the present study showed higher sensitivity and specificity than those cited by current guidelines in men (Table 6). The highest sensitivity and specificity were observed among cut-off points obtained from the 50th percentile for FMI (92% and 83%, respectively, for high LDL-c), followed by BMI (72% for high TC and 49% for high LDL-c, respectively), high WC (67% for high TC and 49% for high LDL-c, respectively), and high BF% (67% for high TC and 48% for high LDL-c, respectively). Only the cut-off point for low FFMI from current guidelines showed higher sensitivity and specificity than those proposed in this study (91% for GLC and 82% for high LDL-c, respectively). Conversely, the cut-off points from current guidelines had the lowest sensitivity and specificity for high WC (20% for high GLC and 1% for high BP, respectively), high BF% (36% for high BP and 17% for high TG, respectively) and high BMI (43% for low HDL-c and 23% for high TG, respectively) compared to those proposed in this study. The cut-off points calculated from averages of ROC curves showed the lowest sensitivity and specificity for high FMI (47% for low HDL-c and 27% for high TG) and low FFMI (47% for low HDL-c and 32% for high BP, respectively).

## 4. Discussion

The clinical use of pre-specified cut-off points for BMI, WC, and BF%, as well as FMI and FFMI, has been recommended to standardize comparisons within and between populations. However, when applied among different populations it causes misclassifications, and a considerable number of subjects, both male and female, may not be classified as obese [26,27]. This is in agreement with our results where lower optimal cut-off values for BMI, BF%, and WC were obtained, in both women and men, compared with those from current guidelines [28,29]. Similarly, the cut-off values for FMI in women and FFMI in men were lower than those given by Peine et al. [30] (FMI: ≥8.4 and ≥6.4 kg/m^2^ for women and men, respectively, and FFMI: ≤16.2 and ≤19.8 kg/m^2^ for women and men, respectively)

Thus far, it is unclear which anthropometric and body composition measurements are better predictors of CVRFs in young people, considering that all adiposity measures are highly heterogeneous according to age, sex, and ethnic group [31]. Hence, the sensitivity and specificity among the cut-off points given by current guidelines and those obtained in the present study, the 50th percentile, and the average of the ROC curve, were compared.

Our results showed that cut-off values from the 50th percentile appear to have a higher discriminatory ability to identify the presence of CVRFs. BMI values of ≥22.1 kg/m^2^ in women and ≥23.3 kg/m^2^ in men, BF% of ≥29.8% in women and ≥19.8% in men, FMI ≥ 6.5 kg/m^2^ in women and ≥ 4.5 kg/m^2^ in men, and WC ≥ 80.3 cm in men especially identify high TG in women, high TC in men, and high LDL-c in both genders.

In contrast, the lowest discriminatory ability was observed when cut-off values from current guidelines for WC, BMI, BF%, and FFMI were used, particularly for the prediction of high TC and low HDL-c in women, and high GLC and high BP in men. This suggests that these cut-off values may underestimate obesity related to CVRFs among Mexican young adults.

Accordingly, it has been reported that the optimal cut-off values of BMI, BF%, WC, and FMI are different from those of current guidelines concerning the presence of CVRFs in different populations (Table 7). The lowest optimal cut-off point for WC was observed among Asian populations such as Japanese (72 and 84 cm in women and men, respectively), Taiwanese (74–83 and 85–87 cm in women and men, respectively), and Singaporean Chinese, Malays, and Indians (75–80 and 80–85 cm in women and men, respectively) [32,33,34]. Concerning BF%, the lowest optimal cut-off points were observed in the Chinese population (21.35% and 23.95% in women and men, respectively) [35]. On the other hand, the lowest optimal cut-off values for BMI were seen in Japanese women (22.5 kg/m^2^) and Omani Arab men (23.2 kg/m^2^) [27,32]. Finally, the lowest cut-off points for FMI were appreciated in Chinese women (7.9 kg/m^2^) and men (7.0 kg/m^2^) [35]. However, it is interesting that the optimal cut-off points for all measures were higher, compared to those obtained in the present study.

It is well-documented that optimal cut-off values vary across different ethnicities due to different factors. First, it has been observed that cut-off values linearly increase with increasing population means. On the other hand, genetic differences play a major role in determining changes in body composition and metabolism in addition to an array of risk factors due to distinct social and environmental factors, such as diet, physical activity, and lifestyle, as well as socioeconomic and demographic status [41,42]. In Mexico, the growth of national income, the increasing urbanization, and the globalization of food production have promoted unhealthy food choices and disincentives to engage in physical activity, leading to a positive energy balance which underlies the increase in the prevalence of overweight and obesity, and negative changes in body composition related to non-communicable diseases, such as CVRFs [43,44].

Our results showed that, compared to all anthropometric and body composition measures, high BMI showed the greatest odds of high TG in women, as well as high WC in men, when cut-off values from current guidelines were included in the logistic regression model [23,24]. When these cut-off points were replaced by those obtained in the present study from the 50th percentile and from the average of ROC curves, high BMI still significantly increased the risk of high TG in women more than the other anthropometric and body composition measures. However, despite high WC continuing to show a significant risk of high TG, high FMI presented the highest risk of TG in men. Thus, Mexican young women with overall obesity and men with elevated fat mass tend to be at greater risk of having CVRFs, especially high TG.

Race-specific characteristics of body composition imply that the relation between obesity and CVRFs may also be different among races [32]. Epidemiological surveys and population health promotion usually take BMI as a useful indicator for measuring whole-body obesity, which is associated with chronic diseases such as dyslipidemia [45,46]. Hu et al. [47] found that a higher BMI was directly associated with higher levels of TG in rural Chinese people aged 20–70 years. Similarly, Knowles et al. [48] reported that Peruvian men and women with high BMI consistently had high odds of having CVRFs, including high TG. The main assumption of the association between BMI and high TG is that body mass, adjusted for stature, is closely associated with body fatness and consequent morbidity and mortality [49]. However, despite this being consistent with our results among women, no such association was observed in men. In recent studies, it has been established that BMI does not always reflect true body adiposity, especially when the value is below 30 kg/m^2^, since BMI is a surrogate marker for body fat and does not take body composition into account [35,50,51].

Chronic exposure to a positive net caloric intake increase genetic predisposition, and sedentary lifestyles are significant contributors to abdominal obesity and dyslipidemias in Mexico [52,53]. It has been established that abdominal obesity, assessed by WC (≥94 and ≥80 cm for men and women, respectively), is a strong predictor of CVRFs like high TG among different populations like Mexicans, Peruvians, Canadians, Brazilians, and Americans [48,54,55,56,57,58]. The most significant contributing factor for obesity-related dyslipidemia is likely uncontrolled fatty acid release from adipose tissue, especially visceral adipose tissue, through lipolysis, which causes an increased delivery of fatty acids to the liver and synthesis of very-low-density lipoprotein (VLDL). Increased levels of free fatty acids can decrease mRNA expression or activity of lipoprotein lipase (LPL) in adipose tissue and skeletal muscle, and the increased synthesis of VLDL in the liver can inhibit the lipolysis of chylomicrons, which promotes hypertriglyceridemia [59].

Accordingly, the present study showed that high WC (≥94 and ≥80 cm for men and women, respectively) significantly increases the risk of high TG, especially in men, since, compared to women, men have consistently been shown to have greater rates of fatty acid release from visceral fat [60]. However, our results showed that the risk of high TG decreased when cut-off points obtained in the present study were used. In contrast, previous studies have shown that the use of specific optimal cut-off points for high WC significantly increases the risk of high TG in populations such as Japanese, Chinese, Iranians, and in rural South Africans [61,62,63,64]. This confirms that the choice of cut-off values for abdominal obesity should be made considering sex, age, and ethnicity to improve the prediction of CVRFs, as has been previously reported [61].

Nevertheless, BMI and WC make the relative contributions of fat and lean tissue in the presence of CVRFs indistinguishable, which makes it possible to observe CVRFs in subjects with BMI and WC considered normal, but who have excess body fat [65,66]. Thus, it has been established that FMI and FFMI should be potentially better indicators for evaluating the relationship between body composition parameters and CVRFs, as well as for determining whether fat or lean tissue is due to height or changes in body composition [67]. However, body composition in young individuals has been rarely studied. Our results showed that whereas men with high FMI had a significantly higher risk of having all CVRFs, especially high TG, low FFMI was associated with the risk of high TG and low HDL- in women and all CVRFs in men. This confirms that adiposity and muscle mass have opposite associations with glucose metabolism and health risk [68].

Regarding FMI, it has been increasingly used as an adiposity index in recent years and is one of the indexes currently employed to evaluate indirect measures of body adiposity [69]. High levels of body fat play a major role in the development of CVRFs, including hypertension, dyslipidemia, and diabetes mellitus, as well as in increasing the risk of developing CVD [70]. It has been recognized that besides storing excess fat, adipose tissue synthesizes and secretes adipokines, including adiponectin, resistin, leptin, plasminogen activator inhibitor-1 (PAI-1), tumor necrosis factor-alpha (TNF-α), and interleukin (IL-6). These adipokines are altered in obesity and are believed to be actively responsible for the insulin resistance state; they are also implicated in the atherosclerotic process in obese individuals [71].

However, obesity not only affects metabolic risk factors but also disrupts skeletal muscle metabolism [72]. It has been demonstrated that obesity is associated with abnormal lipid infiltration within the muscle, the so-called intramuscular adipose tissue (IMAT) [73]. IMAT is associated with diminished muscle insulin sensitivity, through ceramide production and pro-inflammatory adipokines, which contribute to the decline of muscle mass considered an undesirable inherent consequence of physical inactivity [74,75]. Thus, intervention programs that increase levels of physical activity should have protective effects [75]. In this context, a regular exercise program (three times/week) that includes resistance and endurance exercise training would have a major positive effect on improving muscle mass, strength, and function [76].

In agreement with our result, which showed that low FFMI increases the risk of CVRFs, Ferrara et al., Scoot et al., Zhang et al., and Lu et al. reported that low muscle mass or sarcopenia is associated with metabolic syndrome since low muscle mass promotes insulin resistance, hypertriglyceridemia, and the presence of all CVRFs [72,77,78,79,80].

The mechanisms underlying this association may include the fact that muscle accounts for 85% of the body’s glucose disposal [81]. In addition, skeletal muscle cells express and secrete many myokines in response to muscle contracting during physical activity, such as IL-6, IL-8, IL-15, fibroblast growth factor 21, irisin, myonectin, and myostatin. These myokines offset the deleterious effects of inflammatory cytokines and, subsequently, have beneficial effects on glucose and lipid metabolism as well as inflammation [81,82,83].

The major strength of this study is that it could be possible to identify adequate cut-off points for anthropometric measures and their predicted power of anthropometric variables on CVRFs in Mexican young adults. Young adulthood is a novel target group for studying the association between body composition and risk of CVD since in this age group several behavioral and metabolic changes occur leading to the emerging risk factors of CVD [38].

This study also has limitations. First, only regression and not causality could be determined because of the cross-sectional design. The information provided herein is somewhat descriptive and does not provide mechanistic information. Thus, future studies should consider a longitudinal approach to track changes over time and establish causality. Moreover, prospective studies examining the incidence of CVRFs according to our proposed cut-off points are needed to further ascertain the accuracy of our obesity definition in classifying CVRFs. In addition, future studies examining changes in fat and muscle mass will provide further insight into body composition and its participation in a pro-inflammatory state related to the presence of CVRFs. Second, our study did not include lifestyle factors, such as dietary intake and physical activity, as covariates in the risk prediction analysis of CVRFs. Future studies may need to consider lifestyle factors, such as physical activity and dietary habits, to provide more information about their contribution to the variation of optimal cut-off values.

In summary, our study highlights the need for revising current guidelines and considering developing new evidence-based cut-off points to improve the definition of obesity among Mexican young adults and identify those with the greatest risks of CVRFs.

## 5. Conclusions

Lower cut-off points of BMI, WC, BF%, and FMI than those suggested by current guidelines are associated with an increased cardiovascular risk, mainly with high TG. BMI and FMI measurements were identified as the best predictors of CVRFs in Mexican young adults. Both BMI and FMI, easily obtainable and interpretable anthropometric measures, should be included as important tools for the screening of CVRFs among Mexican young adults in clinical practice, and health personnel should apply more appropriate thresholds of these body composition measures than the ones currently recommended.

## Figures and Tables

**Table 1:** Anthropometric and biochemical characteristics of participants according to sex.

	Total	Female	Male	*p*
	(n = 1730)	(n = 848)	(n = 882)	
Age (y)	18.9 ± 0.1	18.9 ± 1.3	18.9 ± 1.4	0.6800
Height (cm)	166.1 ± 0.2	159.7 ± 5.8	172.2 ± 6.2	<0.0001
Weight (kg)	65.1 ± 0.3	58.5 ± 11.1	71.2 ± 13.5	<0.0001
WC (cm)	78.5 ± 0.3	74.8 ± 10.0	82.1 ± 10.7	<0.0001
BF (%)	25.3 ± 0.2	29.9 ± 6.9	20.8 ± 6.7	<0.0001
BMI (kg/m^2^)	23.5 ± 0.1	22.9 ± 3.9	24.0 ± 4.1	<0.0001
FMI (kg/m^2^)	6.1 ± 0.1	7.1 ± 2.8	5.2 ± 2.6	<0.0001
FFMI (kg/m^2^)	17.3 ± 0.1	15.8 ± 1.5	18.66 ± 2.2	<0.0001
SBP (mmHg)	109.1 ± 0.3	104.7 ± 10.2	113.1 ± 11.1	<0.0001
DBP (mmHg)	70.6 ± 0.2	69.0 ± 8.7	72.0 ± 8.9	<0.0001
GLC (mg/dL)	84.9 ± 0.3	83.1 ± 13.9	86.4 ± 14.1	<0.0001
TC (mg/dL)	161.2 ± 0.7	161.5 ± 29	160.9 ± 30.4	0.3877
TG (mg/dL)	103.6 ± 1.5	91.6 ± 46.5	115.0 ± 72.4	<0.0001
HDL-c (mg/dL)	50.9 ± 0.3	54.0 ± 13.8	47.8 ± 11.1	<0.0001
LDL-c (mg/dL)	89.7 ± 0.6	89.1 ± 23.5	90.1 ± 23.9	0.3999

WC, waist circumference; BF%, percentage of body fat; BMI, body mass index; FMI, fat mass index; FFMI, fat-free mass index; BP: blood pressure; GLC, glucose; HDL-c, high-density lipoprotein cholesterol; LDL-c, low-density lipoprotein cholesterol; TG, triglycerides; TC, total cholesterol.

**Table 2:** Optimal cut-points and area under curves for anthropometric and body composition measures for the prediction of cardiovascular risk factors using ROC analysis by sex.

		Women				Men			
		Optimal Cut-Point	Sen (%)	1-Spe (%)	AUC (95% CI)	Optimal Cut-Point	Sen (%)	1-Spe (%)	AUC (95% CI)
WC	BP	86.6	0.2131	0.1073	0.54 (0.48–0.60)	83.0	0.5349	0.3425	0.62 (0.58–0.66)
	GLC	64.6	0.9643	0.8453	0.51 (0.40–0.62)	91.9	0.2917	0.1655	0.56 (0.47–0.64)
	TC	72.8	0.6269	0.4655	0.43 (0.36–0.51)	88.0	0.4938	0.2210	0.65 (0.58–0.72)
	TG	74.5	0.6228	0.3929	0.64 (0.60–0.68)	81.9	0.5831	0.5315	0.64 (0.60–0.67)
	HDL-c	72.1	0.6667	0.4753	0.62 (0.58–0.65)	81.9	0.5402	0.3839	0.59 (0.55–0.63)
	LDL-c	63.4	0.1842	0.1134	0.48 (0.39–0.58)	83.2	0.6327	0.3902	0.61 (0.53–0.69)
	Average	72.3				84.9			
BF%	BP	33.7	0.4672	0.2517	0.62 (0.57–0.68)	20.9	0.5648	0.3752	0.61 (0.57–0.65)
	GLC	28.3	0.7857	0.5907	0.56 (0.45–0.66)	26.1	0.4583	0.1942	0.65 (0.57–0.73)
	TC	26.6	0.7761	0.6816	0.51 (0.44–0.57)	21.9	0.6667	0.3720	0.67 (0.60–0.73)
	TG	32.6	0.5175	0.2754	0.63 (0.59–0.67)	22.2	0.5150	0.2796	0.64 (0.60–0.67)
	HDL-c	29.7	0.6033	0.4424	0.59 (0.55–0.63)	22.8	0.4626	0.2753	0.61(0.57–0.64)
	LDL-c	29.3	0.7105	0.5302	0.58 (0.50–0.66)	21.9	0.6327	0.3854	0.60 (0.51–0.68)
	Average	30				22.6			
BMI	BP	27.3	0.2705	0.1087	0.59 (0.54–0.65)	23.3	0.5548	0.3270	0.64 (0.60–0.68)
	GLC	23.8	0.5000	0.3276	0.54 (0.43–0.65)	24.9	0.5833	0.3261	0.64 (0.57–0.72)
	TC	24.4	0.7761	0.6969	0.51 (0.44–0.57)	25.5	0.5802	0.2734	0.67 (0.61–0.73)
	TG	24.3	0.4912	0.2415	0.65 (0.61–0.69)	23.8	0.5995	0.3476	0.63 (0.60–0.67)
	HDL-c	21.3	0.7080	0.5082	0.62 (0.58–0.66)	24.5	0.4828	0.3015	0.60 (0.56–0.64)
	LDL-c	21.1	0.7895	0.6104	0.58 (0.49–0.66)	24.6	0.5918	0.3481	0.62 (0.54–0.70)
	Average	23.7				24.4			
FMI	BP	8.1	0.4508	0.2655	0.62 (0.56–0.67)	5.0	0.5615	0.3614	0.62 (0.59–0.66)
	GLC	6.5	0.6786	0.4994	0.55 (0.45–0.66)	5.3	0.6458	0.3753	0.65 (0.57–0.73)
	TC	7.5	0.7164	0.6279	0.51 (0.44–0.57)	6.6	0.4815	0.2022	0.67 (0.61–0.74)
	TG	7.6	0.5351	0.2802	0.64 (0.60–0.69)	5.1	0.5749	0.3184	0.64 (0.60–0.68)
	HDL-c	6.7	0.5787	0.4033	0.60 (0.56–0.64)	5.2	0.5144	0.3315	0.61 (0.57–0.65)
	LDL-c	6.4	0.7105	0.5117	0.58 (0.50–0.67)	5.6	0.3361	0.3361	0.61 (0.53–0.69)
	Average	7.1				5.5			
FFMI	BP	15.8	0.5164	0.4237	0.54 (0.48–0.59)	18.9	0.5482	0.3201	0.63 (0.59–0.67)
	GLC	15.5	0.6786	0.5371	0.54 (0.43–0.65)	18.9	0.6042	0.3789	0.60 (0.51–0.68)
	TC	16.8	0.8209	0.7596	0.51 (0.43–0.56)	19.1	0.6049	0.3508	0.63 (0.57–0.70)
	TG	15.8	0.6184	0.3961	0.62 (0.58–0.66)	18.8	0.5395	0.3573	0.60 (0.56–0.64)
	HDL-c	16.1	0.4904	0.2942	0.61 (0.58–0.65)	18.6	0.592	0.4476	0.58 (0.54–0.62)
	LDL-c	16.2	0.5000	0.3402	0.55 (0.46–0.65)	19.1	0.551	0.3481	0.60 (0.52–0.69)
	Average	16				18.9			

Sen, sensibility; Spe, specificity; WC, waist circumference; BF%, percentage of body fat; BMI, body mass index; FMI, fat mass index; FFMI, fat-free mass index; BP: blood pressure; GLC, glucose; HDL-c, high-density lipoprotein cholesterol; LDL-c, low-density lipoprotein cholesterol; TG, triglycerides; TC, total cholesterol. Bold denotes the greatest AUC.

**Table 3:** Age-adjusted odds ratio and 95% confidence intervals for cardiovascular risk factors concerning the diagnosis of anthropometric and body composition measures in young women.

	Model *^,†,‡^	High WC	High BF%	High BMI	High FMI	High FFMI
High BP	1	1.41 (0.93–2.13)	2.54 (1.67–2.85)	2.08 (1.38–3.15)	2.23 (1.48–3.31)	0.89 (0.58–1.36)
	2	1.36 (0.92–2.00)	2.08 (1.39–3.11)	1.61 (1.08–2.38)	2.01 (1.34–3.00)	0.75 (0.51–1.11)
	3	1.28 (0.87–1.90)	2.00 (1.34–2.98)	1.81 (1.22–2.68)	1.95 (1.32–2.89)	0.81 (0.55–1.20)
High GLC	1	1.08 (0.47–2.48)	1.35 (0.59–2.13)	1.19 (0.51–2.74)	1.42 (0.64–3.12)	0.56 (0.22–1.39)
	2	1.16 (0.55–2.48)	1.59 (0.73–3.43)	1.85 (0.84–4.07)	1.84 (0.84–4.03)	0.63 (0.29–1.37)
	3	1.08 (0.50–2.31)	1.69 (0.78–3.65)	1.89 (0.89–4.03)	1.21 (0.57–2.58)	0.73 (0.34–1.55)
High TC	1	0.76 (0.42–1.38)	0.79 (0.42–1.48)	0.76 (0.41–1.41)	0.71 (0.39–1.28)	0.88 (0.51–1.52)
	2	0.57 (0.34–0.96)	1.27 (0.77–2.10)	1.04 (0.63–1.72)	1.10 (0.67–1.82)	1.00 (0.62–1.70)
	3	0.59 (0.36–0.98)	1.19 (0.72–1.96)	0.83 (0.49–1.43)	0.99 (0.60–1.65)	1.24 (0.73–2.10)
High TG	1	3.03 (2.15–4.27)	2.92 (2.03–4.20)	3.27 (2.29–4.66)	3.23 (2.29–4.54)	0.45 (0.31–0.66)
	2	2.69 (1.94–3.73)	2.40(1.74–3.32)	2.87 (2.06–3.99)	2.43 (1.76–3.37)	0.44 (0.32–0.61)
	3	2.69 (1.92–3.77)	2.28 (1.65–3.15)	3.07 (2.21–4.27)	2.85 (2.06–3.95)	0.49 (0.32–0.62)
Low HDL-c	1	1.97 (1.44–2.68)	2.05 (1.48–2.85)	2.07 (1.50–2.84)	2.17 (1.59–2.95)	0.45 (0.33–0.62)
	2	1.96 (1.49–2.59)	1.87 (1.42–2.47)	2.05 (1.55–2.71)	1.99 (1.51–2.64)	0.54 (0.41–0.71)
	3	2.16 (1.62–2.86)	1.86 (1.41–2.45)	2.11 (1.58–2.83)	1.98 (1.50–2.62)	0.46 (0.34–0.61)
High LDL-c	1	1.10 (0.54–2.26)	1.38 (0.67–2.85)	1.57 (0.79–3.13)	1.32 (0.65–2.64)	0.85 (0.41–1.73)
	2	0.90 (0.47–1.73)	2.01 (1.01–3.98)	1.58 (0.81–3.08)	2.25 (1.12–4.53)	0.80 (0.41–1.54)
	3	0.89 (0.46–1.71)	1.90 (0.97–3.72)	1.22 (0.63–2.38)	1.58 (0.82–3.03)	0.62 (0.32–1.19)

WC, waist circumference; BF%, percentage of body fat; BMI, body mass index; FMI, fat mass index; FFMI, fat-free mass index; BP: blood pressure; GLC, glucose; HDL-c, high-density lipoprotein cholesterol; LDL-c, low-density lipoprotein cholesterol; TG, triglycerides; TC, total cholesterol. Bold denotes statistically significant results. * Model 1: included the cut-off points for anthropometric measures given by current guidelines. ^†^ Model 2: included the cut-off points for anthropometric measures consistent with the 50th percentile. ^‡^ Model 3: included the cut-off points for anthropometric measures according to the average determined by the Youden index from ROC curves.

**Table 4:** Age-adjusted odds ratio and 95% confidence intervals for cardiovascular risk factors according to the diagnosis of anthropometric and body composition measures in young men.

	Model *^,†,‡^	High WC	High BF%	High BMI	High FMI	High FFMI
High BP	1	3.30 (2.20–4.96)	2.34 (1.70–3.23)	2.28 (1.90–3.51)	2.30 (1.71–3.08)	0.51 (0.34–0.77)
	2	2.23 (1.66–3.00)	2.08 (1.55–2.78)	2.72 (2.02–3.67)	2.22 (1.65–2.97)	0.41 (0.30–0.55)
	3	2.59 (1.91–3.53)	2.09 (1.55–2.83)	2.64 (1.95–3.55)	2.33 (1.73–3.13)	0.37 (0.27–0.49)
High GLC	1	1.62 (0.79–3.36)	2.72 (1.51–4.90)	2.85 (1.57–5.14)	2.87 (1.56–5.29)	0.37 (0.13–1.05)
	2	1.23 (0.68–2.21)	2.08 (1.12–3.86)	2.19 (1.18–4.08)	2.10 (1.13–3.90)	0.45 (0.24–0.84)
	3	1.26 (0.69–2.30)	2.70 (1.49–4.88)	2.44 (1.35–4.42)	2.45 (1.36–4.44)	0.41 (0.22–0.74)
High TC	1	3.24 (1.94–5.41)	2.83 (1.77–4.52)	3.11 (1.95–4.96)	2.90 (1.80–4.68)	0.59 (0.29–1.21)
	2	2.26 (1.38–3.67)	2.34 (1.43–3.82)	2.52 (1.53–4.16)	2.42 (1.48–3.97)	0.51 (0.32–0.83)
	3	2.44 (1.53–3.88)	2.82 (1.77–4.50)	2.94 (1.83–4.73)	2.82 (1.76–4.52)	0.43 (0.27–0.68)
High TG	1	3.69 (2.44–5.57)	3.36 (2.43–4.63)	3.24 (2.40–4.38)	3.13 (2.34–4.17)	0.68 (0.47–0.98)
	2	2.55 (1.92–3.38)	2.78 (2.10–3.69)	2.75 (2.07–3.67)	2.96 (2.23–3.95)	0.53 (0.40–0.70)
	3	2.78 (2.06–2.74)	2.87 (2.14–3.85)	2.30 (2.24–4.01)	3.05 (2.28–4.08)	0.48 (0.36–0.63)
Low HDL-c	1	2.50 (1.70–3.69)	2.51 (1.84–3.43)	2.26 (1.69–3.03)	2.22 (1.67–2.94)	0.79 (0.55–1.14)
	2	1.77 (1.34–2.33)	1.82 (1.38–2.41)	1.92 (1.45–2.54)	1.95 (1.47–2.57)	0.54 (0.41–0.72)
	3	1.95 (1.46–2.60)	2.33 (1.75–3.11)	2.15 (1.62–2.86)	2.17 (1.63–2.88)	0.54 (0.41–0.72)
High LDL-c	1	1.76 (0.87–3.54)	1.69 (0.93–3.09)	2.61 (1.46–4.68)	2.42 (1.34–4.38)	0.66 (0.27–1.58)
	2	1.77 (0.97–3.21)	1.98 (1.08–3.63)	2.12 (1.15–3.92)	2.14 (1.16–3.96)	0.47 (0.25–0.87)
	3	2.03 (1.13–3.63)	2.35 (1.32–4.22)	2.39 (1.33–4.31)	2.32 (1.29–4.16)	0.55 (0.31–0.98)

WC, waist circumference; BF%, percentage of body fat; BMI, body mass index; FMI, fat mass index; FFMI, fat-free mass index; BP: blood pressure; GLC, glucose; HDL-c, high-density lipoprotein cholesterol; LDL-c, low-density lipoprotein cholesterol; TG, triglycerides; TC, total cholesterol. Bold denotes statistically significant results. * Model 1: included the cut-off points for anthropometric measures given by current guidelines. ^†^ Model 2: included the cut-off points for anthropometric measures consistent with the 50th percentile. ^‡^ Model 3: included the cut-off points for anthropometric measures according to the average determined by the Youden index from ROC curves.

**Table 5:** Sensitivity and specificity of anthropometric and body composition cut-off points for the risk of cardiovascular risk factors in women.

	Cut-Off Point *^,†,‡^	WC		BF%		BMI		FMI		FFMI	
		Sen %	1-Spe %	Sen %	1-Spe %	Sen %	1-Spe %	Sen %	1-Spe %	Sen %	1-Spe %
High BP	1	0.328	0.263	0.377	0.201	0.369	0.229	0.426	0.255	0.705	0.674
	2	0.598	0.539	0.623	0.459	0.459	0.329	0.549	0.397	0.434	0.377
	3	0.557	0.488	0.648	0.472	0.598	0.485	0.639	0.490	0.566	0.499
High GLC	1	0.286	0.270	0.286	0.223	0.286	0.248	0.357	0.277	0.786	0.674
	2	0.571	0.546	0.607	0.478	0.500	0.343	0.464	0.417	0.464	0.383
	3	0.536	0.496	0.607	0.494	0.643	0.496	0.679	0.506	0.607	0.505
High TC	1	0.224	0.274	0.194	0.229	0.209	0.252	0.224	0.284	0.701	0.676
	2	0.433	0.557	0.522	0.479	0.313	0.351	0.418	0.419	0.343	0.389
	3	0.373	0.508	0.552	0.493	0.507	0.501	0.522	0.511	0.493	0.510
High TG	1	0.425	0.213	0.355	0.177	0.399	0.194	0.477	0.218	0.794	0.635
	2	0.706	0.489	0.618	0.432	0.518	0.285	0.583	0.358	0.522	0.335
	3	0.658	0.439	0.645	0.444	0.680	0.435	0.654	0.460	0.654	0.455
Low HDL-c	1	0.345	0.214	0.296	0.173	0.323	0.193	0.365	0.216	0.773	0.607
	2	0.649	0.471	0.569	0.418	0.442	0.278	0.514	0.348	0.494	0.305
	3	0.591	0.428	0.586	0.432	0.605	0.424	0.605	0.442	0.597	0.442
High LDL-c	1	0.289	0.272	0.289	0.223	0.342	0.244	0.342	0.277	0.711	0.677
	2	0.526	0.548	0.632	0.475	0.395	0.346	0.526	0.414	0.500	0.380
	3	0.474	0.499	0.658	0.49	0.605	0.496	0.684	0.504	0.553	0.506

Sen, sensibility; Spe, specificity; WC, waist circumference; BF%, percentage of body fat; BMI, body mass index; FMI, fat mass index; FFMI, fat-free mass index; BP: blood pressure; GLC, glucose; HDL-c, high-density lipoprotein cholesterol; LDL-c, low-density lipoprotein cholesterol; TG, triglycerides; TC, total cholesterol. Bold denotes the greatest sensibility and specificity. * Cut-off points for anthropometric measures given by current guidelines. ^†^ Cut-off points for anthropometric measures consistent with the 50th percentile. ^‡^ Cut-off points for anthropometric measures according to the average determined by the Youden index from ROC curves.

**Table 6:** Sensitivity and specificity of anthropometric and body composition cut-off points for the risk of cardiovascular risk factors in men.

	Cut-Off Point *^,†,‡^	WC		BF%		BMI		FMI		FFMI	
		Sen %	1-Spe %	Sen %	1-Spe %	Sen %	1-Spe %	Sen %	1-Spe %	Sen %	1-Spe %
High BP	1	0.229	0.096	0.362	0.212	0.452	0.267	0.522	0.343	0.877	0.795
	2	0.468	0.289	0.452	0.305	0.525	0.317	0.485	0.315	0.548	0.322
	3	0.615	0.442	0.598	0.446	0.651	0.432	0.631	0.454	0.645	0.448
High GLC	1	0.208	0.138	0.479	0.251	0.563	0.317	0.646	0.390	0.917	0.818
	2	0.396	0.348	0.583	0.342	0.583	0.376	0.583	0.361	0.604	0.387
	3	0.563	0.499	0.667	0.488	0.688	0.496	0.667	0.506	0.688	0.505
High TC	1	0.321	0.122	0.469	0.242	0.580	0.305	0.630	0.381	0.889	0.816
	2	0.543	0.331	0.580	0.332	0.630	0.363	0.605	0.350	0.605	0.378
	3	0.679	0.481	0.679	0.479	0.728	0.484	0.704	0.496	0.679	0.498
High TG	1	0.223	0.083	0.390	0.173	0.466	0.233	0.548	0.301	0.850	0.804
	2	0.466	0.268	0.482	0.256	0.523	0.291	0.512	0.274	0.496	0.330
	3	0.627	0.412	0.629	0.404	0.638	0.414	0.651	0.417	0.591	0.460
Low HDL-c	1	0.210	0.097	0.368	0.195	0.431	0.264	0.514	0.331	0.839	0.813
	2	0.431	0.298	0.457	0.273	0.491	0.320	0.474	0.307	0.477	0.348
	3	0.583	0.448	0.580	0.444	0.595	0.449	0.606	0.455	0.595	0.463
High LDL-c	1	0.224	0.136	0.367	0.257	0.531	0.318	0.868	0.721	0.878	0.820
	2	0.510	0.341	0.551	0.343	0.592	0.376	0.842	0.684	0.551	0.390
	3	0.633	0.493	0.653	0.489	0.694	0.496	0.921	0.835	0.694	0.504

Sen, sensibility; Spe, specificity; WC, waist circumference; BF%, percentage of body fat; BMI, body mass index; FMI, fat mass index; FFMI, fat-free mass index; BP: blood pressure; GLC, glucose; HDL-c, high-density lipoprotein cholesterol; LDL-c, low-density lipoprotein cholesterol; TG, triglycerides; TC, total cholesterol. Bold denotes the greatest sensibility and specificity. * Cut-off points for anthropometric measures given by current guidelines. ^†^ Cut-off points for anthropometric measures consistent with the 50th percentile. ^‡^ Cut-off points for anthropometric measures according to the average determined by the Youden index from ROC curves.

**Table 7:** Accurate cut-off values of BMI, BF%, WC, and FMI, concerning the presence of CVRFs in different populations.

Author	CVRFs Included	Population	WC (cm)	BF% (%)	BMI (kg/m^2^)	FMI (kg/m^2^)
Macias et al. 2014 [18]	SBP, GLC, TG, HDL-c	Mexican adults 20–65 years	W = 86–91 M = 92–94	W = 42.3–44.0 M = 29.6–30.5	W =25.3–27.2 M = 26.3–27.2	
Ito et al. 2003 [32]	BP, GLC, TC, TG, glycated hemoglobin	Japanese adults 20–79 years	W = 72 M = 84	W= 35.0 M= 24.0	W = 22.5 M = 23.5	
Tseng et al. 2010 [33]	BP, GLC, TG, HDL-c	Taiwanese adults 25–75 years	W = 74–83 M = 85–87		W = 22.10–23.21 M = 23.74–26.26	
Lin et al. 2002 [36]	BP, GLC, TC, TG, HDL-c, LDL-c	Taiwanese adults W= 37.0 ± 11.1 years M= 37.3 ± 10.9 years	W = 71.5 M = 80.5		W = 22.1 M = 23.6	
Liu et al. 2013 [35]	GLC, TC, TG, HDL-c, LDL-c, CRP	Chinese adults 20–79 years	W = 71.5 M = 80.5	W = 21.35 M = 23.95	W = 23.85 M = 27.45	W = 7.9 M = 7.0
Deurenberg-Yap et al. 2002 [34]	BP, GLC, TC, TG	Singaporean Chinese, Malays, and Indians 18–75 years	W = 75.0–80.0 M = 80.0–85.0		W = 24.0 M0 24.0	
Rodrigues et al. 2023 [37]	BP, GLC, TC, TG, HDL-c, LDL-c, CRP, glycated hemoglobin	Brazilian adults Group 1 = 30 years Groups 2 = 37–39 years		Group 1: W = 37.4–39.7 M = 25.2–27.8 Groups 2: W = 38.5–42.2 M = 26.1–27.8	Group 1: W = 25.4–27.2 M = 26.3–27.3 Groups 2: W = 27.2–29.6 M = 28.3–29.0	Groups 1: W = 9.5–10.8 M = 6.3–7.5 Groups 2: W = 10.2–12.2 M = 7.3–7.8
Ramírez-Vélez et al. 2017 [38]	BP, GLC, TC, TG, HDL-c, LDL-c	Colombian adults 18–35 years		W= 38.9 M= 25.5		W = 11.8 M = 6.9
Al-Lawati et al. 2008 [27]	BP, GLC, TG, HDL-c	Omani Arab adults ≥20 years	W = 84.5 M = 80.0		W = 26.8 M = 23.2	
Głuszek et al. 2020 [39]	BP, GLC, TG, HDL-c	Polish Caucasian 55.7 ± 5.4 years	W = 87.0 M = 97.0		W = 27.2 M = 27.1	
Raposo et al. 2018 [40]	BP, GLC, TC, TG, HDL-c, CRP	Portuguese adults ≥18 years	W = 89.0 M = 93.5		W = 26.5 M = 27.0	

WC, waist circumference; BF%, percentage of body fat; BMI, body mass index; FMI, fat mass index; W, women; M, men; BP: blood pressure; GLC, glucose; HDL-c, high-density lipoprotein cholesterol; LDL-c, low-density lipoprotein cholesterol; TG, triglycerides; TC, total cholesterol; CRP, C-reactive protein.

## Data Availability

The data presented in this study are available on request from the corresponding author. The data are not publicly available due to privacy and ethical restrictions.
